# Exploring Species Limits in Two Closely Related Chinese Oaks

**DOI:** 10.1371/journal.pone.0015529

**Published:** 2010-11-30

**Authors:** Yan-Fei Zeng, Wan-Jin Liao, Rémy J. Petit, Da-Yong Zhang

**Affiliations:** 1 Key Laboratory of Silviculture of the State Forestry Administration, Research Institute of Forestry, Chinese Academy of Forestry, Beijing, China; 2 Ministry of Education Key Laboratory for Biodiversity Science and Ecological Engineering, College of Life Sciences, Beijing Normal University, Beijing, China; 3 INRA, UMR Biodiversity, Genes and Ecosystems, Cestas, France; 4 University of Bordeaux, UMR Biodiversity, Genes and Ecosystems, Cestas, France; University of Umeå, Sweden

## Abstract

**Background:**

The species status of two closely related Chinese oaks, *Quercus liaotungensi*s and *Q. mongolica*, has been called into question. The objective of this study was to investigate the species status and to estimate the degree of introgression between the two taxa using different approaches.

**Methodology/Principal Findings:**

Using SSR (simple sequence repeat) and AFLP (amplified fragment length polymorphism) markers, we found that interspecific genetic differentiation is significant and higher than the differentiation among populations within taxa. Bayesian clusters, principal coordinate analysis and population genetic distance trees all classified the oaks into two main groups consistent with the morphological differentiation of the two taxa rather than with geographic locations using both types of markers. Nevertheless, a few individuals in Northeast China and many individuals in North China have hybrid ancestry according to Bayesian assignment. One SSR locus and five AFLPs are significant outliers against neutral expectations in the interspecific *F*_ST_ simulation analysis, suggesting a role for divergent selection in differentiating species.

**Main Conclusions/Significance:**

All results based on SSRs and AFLPs reached the same conclusion: *Q. liaotungensi*s and *Q. mongolica* maintain distinct gene pools in most areas of sympatry. They should therefore be considered as discrete taxonomic units. Yet, the degree of introgression varies between the two species in different contact zones, which might be caused by different population history or by local environmental factors.

## Introduction

Natural hybridization occurs frequently in plants and animals [1]. Analysis of natural hybridization and hybrid zones provides insight into the processes of introgression, speciation and reproductive isolation [1]–[4]. While contemporary hybridization and introgression have long been thought to threaten species persistence, more recent work suggests that these processes are not necessarily a major impediment to effective species delimitation [5]. However, they can lead to species barriers of varying strength across different contact zones, a feature of great potential interest to understand the evolution of reproductive isolation as well as its breakdown [6], [7].

The oaks (*Quercus*) should be good models to evaluate the effects of hybridization and introgression on species delimitation. They have long been recognized as a challenge to the ideal standard of discrete biological species because of their propensity to intercross [8]–[10]. Morphologically intermediate forms are frequently observed [11], [12]. Such intermediate forms can be especially abundant locally [e.g. 13]. These populations are then called hybrid swarms, which are defined as “an extremely variable mixture of species, hybrids, backcrosses, and later-generation recombination types” [10].

Earlier studies on oaks have shown that chloroplast (cp) DNA haplotypes are often shared in areas of sympatry [14]–[16]. They also established that sibling species pairs are more distinctly discriminated by morphological or ecological (i.e., adaptive) traits than by isozyme or nuclear DNA markers [17]–[20]. However, in contrast to cpDNA markers, some genetic differentiation generally exists at nuclear DNA markers [e.g. 17], [ 18], [ 20], [ 21]. These studies also provided the first quantitative evidence that the strength of species barriers can vary geographically [22], [23]. More recent studies relying on a combination of powerful markers such as SSRs (in sufficient number) or AFLPs in combination with effective assignment methods have successfully distinguished among closely related oak species [24]–[28]. Individuals can generally be assigned to their respective species using their multilocus genotypes irrespective of their physical appearance, providing evidence for bimodal distribution of characters, with few individuals with intermediate characters and many parental types [25], [29]. For instance, Muir and Schlötterer [30] found that all studied individuals of *Q. petraea* and *Q. robur*, two closely related European oak species that have been extensively studied, can be assigned to either of the two species. This led them to call into question the importance of hybridization in these species, thereby resurrecting an old controversy [31]. However, subsequent studies relying on SSR markers confirmed that there are evolutionary significant rates of hybridization between *Q. robur* and *Q. petraea* and between other oak species pairs [32]–[37], confirming earlier work [12].

In China, problems of taxonomic discrimination occur between the Liaotung oak (*Q. liaotungensis* Koidz.) and the Mongolian oak (*Q. mongolica* Fisch. ex Lede). Taxonomists distinguish the two species by subtle morphological differences: *Q. liaotungensis* has smooth-cupule acorns and 5–7 pairs of lateral veins per leaf, whereas in *Q. mongolica* the acorn cupule is rough and there are 8–12 pairs of lateral veins per leaf [38]. However, due to the morphological plasticity of oaks and the potential for hybridization in sympatric populations, these characters can be confusing. For instance, different local floras record different ranges of variation in number of lateral veins within each species, such as 7–9, 7–10, 7–13 and 8–10 for *Q. mongolica* and 5–7, 5–8 and 5–9 for *Q. liaotungensis* [39]. The Chinese version of *Flora of China* [40], *Higher Plants of China* [38] and many local floras discriminate the two species, but the English version of *Flora of China* [41] considers them as belonging to the same species.

In an attempt to elucidate whether the morphologically identified *Q. liaotungensis* and *Q. mongolica* are genetically distinct from each other, we examine genetic variation in 419 individuals from 15 oak populations with both SSR and AFLP markers. We also investigate if the markers are equally powerful at delimiting taxa or if there is some heterogeneity among loci or marker type. Finally, we evaluate to what extent species boundaries are homogeneous across the sympatric range or instead vary in strength geographically. To answer these questions, we rely on conventional population genetic analysis as well as on a Bayesian clustering approach without consideration of sampling locations and taxonomic status.

## Materials and Methods

### Study species

According to the description of *Higher Plants of China* [38], *Quercus mongolica* Fisch. ex Ledeb. is a common tree species of temperate, low-elevation broadleaved woodlands, with a widespread distribution in North China, Northeast China and in parts of Russia, North Korea and Japan; *Q. liaotungensis* Koidz. ( = *Q. wutaishanica* Mayr) is another dominant broadleaved tree in the warm temperate zone, with a main distribution in northern China and partially in North Korea [38]. Recently, *Q. liaotungensis* has also been reported in the Russian Far East [42]. In accordance with their ecological amplitudes, the two species can occur in the same locality. They have similar reproductive biology, characterized by monoecy, anemophily and seed dispersal by gravity and animals.

### Sampling design

**Figure 1:**
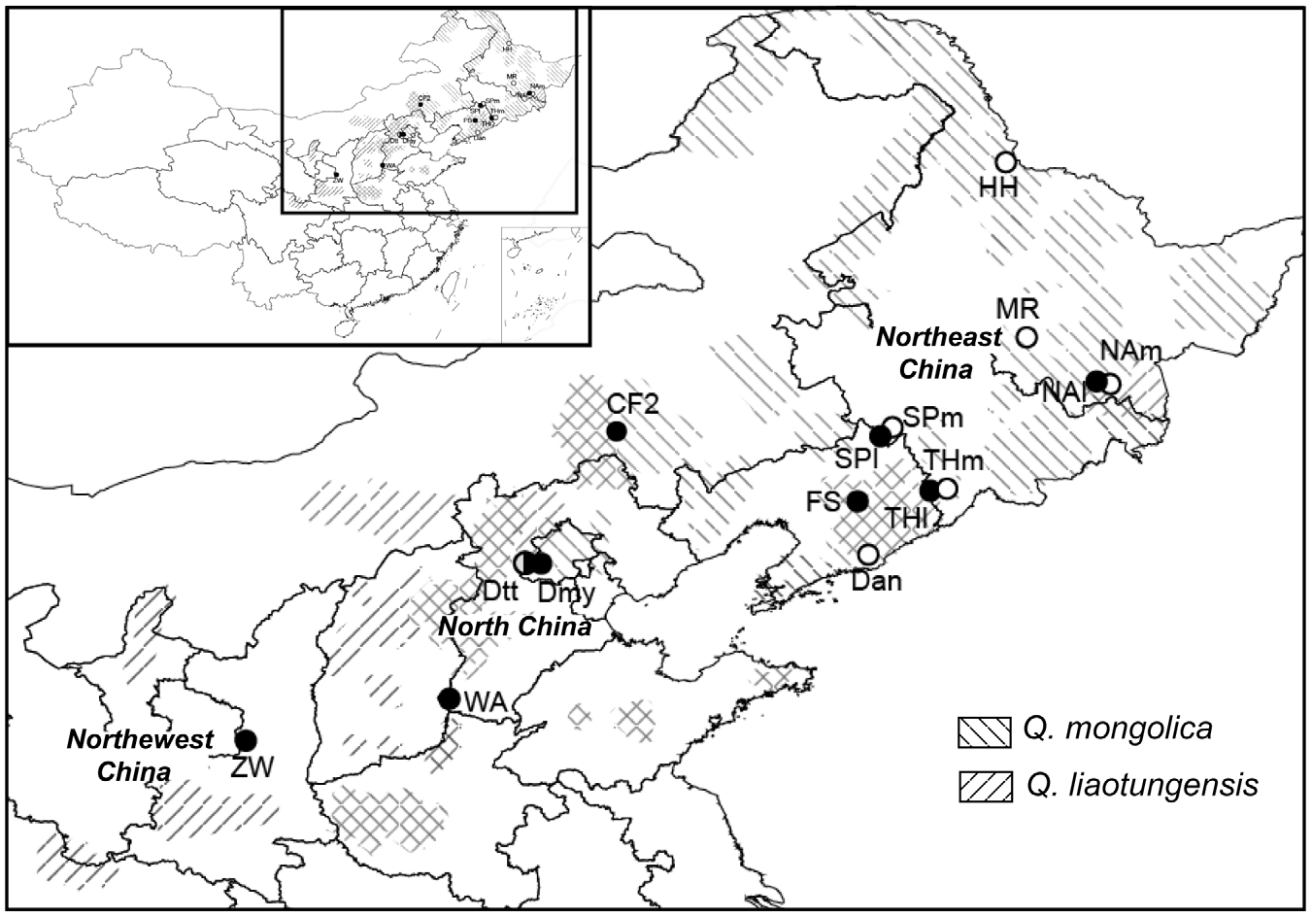
Geographic distribution of *Quercus liaotungensis* and *Q. mongolica* in China and location of populations sampled. Shadows show the distribution of *Q. liaotungensis* and *Q. mongolica*, respectively, according to Zhang [38]. Filled circles: *Q. liaotungensis*; open circles: *Q. mongolica*; half filled circles: intermediate populations.

Leaf samples were collected from 419 individuals from a total of 15 oak populations across their distribution range in China (Table 1 and Fig. 1). Individuals were identified in the field as *Q. liaotungensis* (smooth acorn cupule), or *Q. mongolica* (rough acorn cupule), following *Higher Plants of China* [38]. Our observations suggest that *Q. mongolica* has smoother trunk bark than *Q. liaotungensis*. Therefore, the character of trunk bark was also considered where acorn cupule morphology alone was not sufficient to differentiate oak individuals (population THl and THm from Tonghua, and population SPl and SPm from Siping). According to above rules, four ‘pure’ *Q. liaotungensis* populations were sampled. Populations ZW and WA are located in areas where *Q. mongolica* is completely absent (allopatric range); CF2 and FS are located in areas from the sympatric range where *Q. mongolica* is locally absent. Three pure *Q. mongolica* populations were also collected. Populations HH and MR are located in areas where *Q. liatungensis* is completely absent (allopatric range); Dan is within the sympatric range in an area where *Q. mongolica* is locally absent. At three locations both species were found within mixed forests. Individuals were randomly sampled and then categorized into different species as far as possible, thus resulting in three pairs of populations: SPl and SPm, THl and THm, and NAl and NAm, where the lower-case letter ‘l’ represents *Q. liaotungensis* and ‘m’ *Q. mongolica*. In addition, one *Q. liaotungensis*-type population (Dmy) and one morphologically intermediate population (Dtt) were sampled from the Dongling Mountain region where controversy has arisen among Chinese taxonomists because of the existence of a broad array of morphologically intermediate trees [43]. These last two populations were excluded from the diversity and differentiation analyses due to the dominance of intermediates in Dtt and too small population sizes for Dmy. Leaf tissues (1–3 leaves per tree) were collected from each sampled tree, dried with silica gel and taken back to the laboratory.

**Table 1:** Description of 15 *Quercus* populations analyzed.

Population type	Sampling Location	Population ID	Species	Longitude (E)	Latitude (N)	Sample size	*H_E_*	*H_j_*
Pure site	Ziwuling, Shaanxi	ZW	*Q. liaotungensis*	108°59′	35°30′	31	0.771	0.207
	Wu'an, Hebei	WA	*Q. liaotungensis*	113°47′	36°55′	30	0.769	0.213
	Chifeng, Neimenggu	CF2	*Q. liaotungensis*	117°58′	43°18′	30	0.764	0.229
	Fushun, Liaoning	FS	*Q. liaotungensis*	124°15′	41°50′	29	0.772	0.204
	Heihe, Heilongjiang	HH	*Q. mongolica*	127°19′	50°11′	29	0.713	0.214
	Mao'ershan, Heilongjiang	MR	*Q. mongolica*	127°40′	45°24′	32	0.724	0.205
	Dandong, Liaoning	Dan	*Q. mongolica*	124°10′	40°18′	28	0.760	0.211
Mixed site	Siping, Jilin	SPl	*Q. liaotungensis*	124°15′	43°13′	26	0.797	0.219
		SPm	*Q. mongolica*	124°15′	43°13′	29	0.793	0.223
	Tonghua, Jilin	THl	*Q. liaotungensis*	125°55′	41°38′	30	0.730	0.212
		THm	*Q. mongolica*	125°55′	41°38′	30	0.757	0.211
	Ning'an, Heilongjiang	NAl	*Q. liaotungensis*	129°32′	44°21′	25	0.785	0.234
		NAm	*Q. mongolica*	129°32′	44°21′	26	0.765	0.213
Intermediate rich site	Dongling Mountain, Beijing	*Dmy	*Q. liaotungensis*	115°27′	39°58′	12	0.776	0.232
		*Dtt	Intermediate	115°26′	39°57′	32	0.772	0.225

*Populations that were eliminated from the diversity and differentiation comparison between species due to small population sizes or intermediate morphology; *H_E_*: mean expected heterozygosity across the 19 SSR loci; *H_j_*: unbiased estimates of genetic diversity (analogous to *H_E_*) based on the 194 AFLP markers.

### Marker analysis and scoring

Total genomic DNA was extracted from 25 mg of leaf tissue from each individual oak and purified using a Plant Genomic DNA Extraction Kit (Tiangen, Beijing, China). The oak samples were screened for variation at 19 nuclear SSR loci that had been developed for other oak species [44]–[49] (see Table 2 for primer details). PCR amplification with each primer pair was performed separately with a PTC-200 thermal cycler (MJ Research Inc.) in a 15-µL reaction volume. The PCR reaction mixture contained 10 mM Tris-HCl (pH 8.0), 1.5 mM MgCL_2_, 200 µM dNTP, 0.3 µM (each) primer, 20 ng of DNA template, and 0.5 U *Taq* polymerase (TaKaRa Company, Tokyo, Japan). PCR amplifications were performed as follows: an initial denaturation step at 94°C for 4 min followed by 31 cycles of 45 s at 94°C, 45 s at an annealing temperature and 45 s at 72°C, and a final extension step at 72°C for 8 min.

**Table 2:** Description and reference of the 19 SSR loci analyzed in current study.

Locus	Motif	*T_a_* (°C)	Allele range (bp)	Label^a^	Reference
ssrQpZAG36	(AG)_19_	50	206–246	HEX	[46]
ssrQpZAG16	(AG)_21_	58	137–177	HEX	[46]
ssrQpZAG15	(AG)_23_	50	102–150	FAM	[46]
ssrQpZAG110	(GA)_14_	50	190–244	FAM	[46]
quru-GA-0C11	(GA)_11_	53	190–248	TAMRA	[48]
quru-GA-0C19	(CT)_7_	53	193–227	FAM	[48]
quru-GA-0M05	(GA)_15_	53	182–240	TAMRA	[48]
quru-GA-0M07	(GA)_19_	48	180–252	TAMRA	[48]
quru-GA-1C08	(AG)_10_	52	251–289	HEX	[48]
MSQ4	(AG)_12_	48	191–269	FAM	[44]
MSQ13	(GA)_29_	50	190–246	HEX	[44]
MSQ16	(GA)_7_	55	178–232	TAMRA	[45]
ssrQrZAG7	(TC)_17_	50	117–169	TAMRA	[47]
ssrQrZAG87	(TC)_20_	50	97–185	HEX	[47]
ssrQrZAG96	(TC)_20_	53	133–195	FAM	[47]
ssrQrZAG101	(TC)_20_(AG)_15_	52	127–181	FAM	[47]
ssrQrZAG102	(GA)_29_	52	193–309	FAM	[47]
ssrQrZAG112	(GA)_32_	52	73–115	TAMRA	[47]
bcqm42	(GT)_11_	52	104–146	HEX	[49]

a Forward primers were modified at the 5′ end with a fluorescent label: HEX (green), 6-FAM (blue), or TAMRA (yellow) (see the Materials and Methods, PCR amplification).

*T_a_*: annealing temperature.

AFLP fingerprinting was performed according to the original protocol of Vos *et al*. [50] except that digestion and ligation were carried out simultaneously. In brief, 50–500 ng of DNA was digested for 3 h at 37°C with 8 U *EcoR*I and 2 U *Mse*I in 20 µL 1×NEB buffer (10 mM Tris-HCl pH 7.5, 1 mM MgCL_2_, 5 mM NaCL, 0.0025% Triton X-100) with 2 µg/ml BSA. Simultaneously, two adaptors, one for the *EcoR*I ends and one for the *Mse*I ends, were ligated to cutting sites by adding 0.2 µM adapters, 80 U T4 DNA ligase and 1 mM ATP within the digesting mixture. Selective pre-amplification was performed with primers (E01 = 5′-GACTGCGTACCAATTCA-3′ and M02 = 5′-GATGAGTCCTGAGTAAC-3′) complementary to adapters, but with one base extension. Each PCR was performed in a 20-µL reaction volume using 2 µL ligated product, primer concentration 0.25 µM, 10 mM Tris-HCl (pH 8.0), 1.5 mM MgCl_2_, 0.2 mM dNTP each and 0.5 U *Taq* DNA polymerase (TaKaRa Company, Tokyo, Japan). The selective pre-amplification was carried out in a PTC-200 thermal cycler (MJ Research Inc.) with the following thermo-cycling parameters: an initial denaturation step at 94°C for 2 min followed by 28 cycles of denaturation at 94°C for 45 s, annealing at 56°C for 45 s, extension at 72°C for 1 min, and a final extension step at 72°C for 10 min. The quality and quantity of the pre-amplified products obtained were determined on 1.0% agarose gels and diluted (1∶19) with ddH_2_O. The selective amplification was performed with four primer combinations (AGA/CTC, AGT/CTC, AAC/CTC, AAC/CAG). Each PCR was performed in a 20-µL reaction volume using 3 µL diluted pre-amplification product, 0.25 µM E-primer labeled fluorescence with 6-FAM (Sangon, Shanghai, China), 0.3 µM M-primer, 10 mM Tris-HCl (pH 8.0), 1.5 mM MgCl_2_, 0.2 mM dNTP and 0.5 U *Taq* DNA polymerase (TaKaRa Company, Tokyo, Japan). Amplification was performed with a touch down cycling process: an initial denaturation step at 94°C for 2 min, then 1 cycle of 30 s at 94°C, 30 s at 65°C, 1 min at 72°C, followed by 11 cycles in which the annealing temperature decreases 0.7°C per cycle, followed by 22 cycles of 30 s at 94°C, 30 s at 56°C and 1 min at 72°C, and a final extension step at 72°C for 5 min.

The SSR genotyping of all individuals was performed by assessing allele size on an ABI 3100 automated Genetic Analyzer (Applied Biosystems), using forward primers labeled with 6-FAM, HEX, or TAMRA (Sangon, Shanghai, China) and the ROX 500 (Applied Biosystems) as an internal standard. Allele sizing was performed using the GENEMAPPER software version 3.7 (Applied Biosystems). In the AFLP analysis, selective PCR products were also separated on an ABI 3100 automated sequencer (Applied Biosystems) with a genescan ROX 500 internal size standard. Electropherograms were subsequently analyzed using GENEMAPPER software version 3.7 (Applied Biosystems). The intensity of each individual peak was normalized on the basis of the total signal intensity and the peak was considered only if its intensity exceeded a fixed threshold. The multilocus AFLP profiles were scored as present (1), absent (0) or ambiguous (?) to create binary matrices. Each set of 48 reactions included a positive (known genotype) and a negative (water) control carried from restriction digest through to the final automated sequencer analysis for AFLP and from PCR through to the final automated sequencer analysis for SSR. Allele size determinations were performed twice manually to reduce scoring errors.

### Genetic diversity analysis

Descriptive statistics for SSR such as the number of alleles, allele frequencies, observed and expected heterozygosities (*H_O_* and *H_E_*) were calculated using the program FSTAT 2.9.3 [51]. We used the software GENEPOP 4.0 for Windows [52] to test for homogeneity of allele distributions between species. We also counted the number of private alleles for each species.

For AFLP, percentage of polymorphic loci (5% level), unbiased estimates of genetic diversity (*H_j_*, analogous to *H_E_*) and differentiation statistics were calculated using the AFLP-SURV 1.0 software [53]. With this software, allelic frequencies at AFLP loci were calculated from the observed frequencies of fragments using the Bayesian approach proposed by Zhivotovsky [54] for diploid species. A non-uniform prior distribution of allelic frequencies was assumed with its parameters derived from the observed distribution of fragment frequencies among loci [54]. These allelic frequencies were used as the input for the analysis of genetic diversity within and between samples following the method described in Lynch and Milligan [55].

### Genetic differentiation

The significance of the genetic differentiation between species or among populations within species was tested by comparison of the observed *F*_ST_ with a distribution of *F*_ST_ under the hypothesis of no genetic structure, obtained by means of 5,000 random permutations of individuals between species or among populations. The *F*_ST_ analogue *θ* of Weir and Cockerham [56] was calculated for the 19 SSR loci using the program FSTAT 2.9.3 [51]. For AFLP, *F*_ST_ was calculated using the AFLP-SURV 1.0 software [53]. This program uses the approach of Lynch and Milligan [55] to calculate population genetic parameters on the basis of the expected heterozygosity of dominant marker loci.

Genetic differentiation of population pairs was estimated with *θ* of Weir and Cockerham [56] for the SSR loci and *Φ*_PT_ (a statistic analogous to *F*_ST_ [57]) for AFLPs. *θ*-values with significance level (obtained by bootstrapping loci 10,000 times) were calculated in FSTAT 2.9.3 [51] and *Φ*_PT_-values with significance level (obtained by 999 times permutation) were calculated in GenAlEx6 [58].

Genetic distances, *D_S_* [59] for SSR and *D* [60] modified for dominant markers by Lynch and Milligan [55] for AFLP, were calculated for each population pair using the programs GENDIST in the PHYLIP package [61] for SSR and AFLP-SURV 1.0 [53] for AFLP. The UPGMA (Unweighted Pair Group Method with Arithmetic mean) trees were generated using the program NEIGHBOR in the PHYLIP package [61] and bootstrapped 1000 times.

A principal coordinate analysis (PCo) was performed for both AFLP and SSR data sets to calculate principal co-ordinates from pairwise Euclidian distance estimates between individual genotypes. Analyses were executed in GenAlEx6 [58]. The first two axes were plotted graphically with Origin 7.5 (OriginLab, Northampton, MA).

### Bayesian admixture analysis

A model-based clustering method implemented in the program STRUCTURE version 2.2 [62]–[64] was used to determine the probability of each individual (non-admixture models) or the proportion of each individual's genome (admixture model) from homogenous clusters without consideration of sampling locations for both the SSR and the AFLP data sets. Estimated posterior probabilities for the simulated model fitting the data were calculated for all samples assuming a uniform prior for *K* (number of possible clusters), and every cluster pattern from 1 to 10 was simulated. After fitting both the admixture and non-admixture models and an initial test of varying the burn-in and run length, ten replicates for each *K* were analyzed using the following parameters: we assumed correlated allele frequencies and an admixed origin of populations; burn-in was set to 100,000 with 1,000,000 additional cycles. STRUCTURE output, Pr(X|K), can be used as an indication of the most likely number of groups. In addition, following Evanno *et al.* [65], *ΔK*, where the modal value of the distribution is located at the real *K*, was also calculated using the software Structure2[1].2-sum (supplied by Dorothée Ehrich). For graphic visualization of the STRUCTURE results, we used DISTRUCT [66].

For the most likely STRUCTURE run (*K* = 2; see Results), the approximate Bayes factors (ratio of the estimated marginal likelihood of the admixture model to that of the non-admixture model) favoring admixture were 1.1×10^36^ (−33651.2 vs −33734.2) and 7.7×10^131^ (−41280.9 vs −41584.5) for SSR and AFLP, respectively. Under the admixture model, the posterior probability approximates the proportion of each individual's genome that is derived from each species [62]. The individuals were assigned to pure species or hybrid categories according to the estimated posterior probability at *K* = 2. A threshold value 0.8 was used as a compromise between efficiency and accuracy [67]. The percentage of hybrid oaks was compared among Siping, Tonghua, Ning'an and Dongling Mountain region where the two species and hybrids co-occur, using Chi-square tests with the crosstabs analysis in SPSS (SPSS Inc., Chicago IL).

### Identification of loci under selection

To address the question of whether adaptive divergence occurs between *Q. liaotungensis* and *Q. mongolica*, we used a coalescent simulation outlined in Beaumont and Nichols [68] to identify ‘outlier loci’ whose empirically derived levels of differentiation place them at the upper extreme of simulated distributions of differentiation based on neutral loci. The program FDIST2 was used to simulate a null distribution of *F*_ST_ values (conditional on heterozygosity) for the SSR data set under an infinite-alleles model and a symmetrical two-island model of population structure. Simulations employed the median of observed *F*_ST_ and the same sample sizes as used in the empirical study. Outlier loci were detected by comparing the empirical distribution of *F*_ST_'s with a simulated distribution derived from 50,000 paired values of *F*_ST_ and heterozygosity at 99th quantiles. The significant AFLP outlier loci were identified by plotting *F*_ST_ against heterozygosity under the assumption of Hardy-Weinberg equilibrium using the program Dfdist [68], [69]. Loci with *F*_ST_ values that fell outside the 99th quantiles threshold were obtained by generating a null distribution of *F*_ST_-values based on 50,000 simulated loci with a mean *F*_ST_ equivalent to ‘neutral’ mean *F*_ST_ of empirical distribution, which was obtained by trimming the 30% highest and lowest *F*_ST_ values.

## Results

### Genetic diversity and differentiation

All SSR loci studied were highly variable, with 5–45 alleles per locus (21 on average) and a total of 406 alleles found across all loci in our sample of 419 individuals. The mean expected heterozygosity (*H_E_*) across all loci for each population varied from 0.713 to 0.797 (Table 1). The number of alleles, observed heterozygosity (*H_O_*), expected heterozygosity (*H_E_*) across all loci were similar between *Q. liaotungensis* and *Q. mongolica*. However, there were some differences between species at some loci, e.g., ssrQpZAG15, ssrQpZAG110, quru-GA-0C11, ssrQrZAG87, and ssrQrZAG112 (Table 3). *Q. liaotungensis* and *Q. mongolica* shared most frequent alleles. Nevertheless, species-specific alleles were found at several loci, especially rare alleles restricted to *Q. mongolica* (Table 3). In fact, none of these private alleles had a frequency higher than 4%. Tests for heterogeneity of allele distributions were highly significant for all 19 SSR loci (*P*<0.0001).

**Table 3:** Comparison of genetic diversity and differentiation between *Quercus liaotungensis* and *Q. mongolica* based on the 19 SSR loci.

	N		*A_n_*		*A_P_*		*H_O_*		*H_E_*		*θ*		
Locus	QL	QM	QL	QM	QL	QM	QL	QM	QL	QM	Between species	QL	QM
ssrQpZAG36	201	174	13	14	1	2	0.677	0.743	0.746	0.823	0.158	0.074	0.030
ssrQpZAG16	201	174	19	18	1	0	0.851	0.878	0.903	0.914	0.008	0.041	0.024
ssrQpZAG15	201	174	18	13	7	2	0.555	0.242	0.605	0.240	0.081	0.043	0.031
ssrQpZAG110	201	173	15	21	1	7	0.680	0.542	0.646	0.595	0.103	0.055	0.022
quru-GA-0C11	201	174	20	20	1	1	0.832	0.615	0.895	0.634	0.132	0.066	0.017
quru-GA-0C19	201	173	5	5	0	0	0.405	0.287	0.395	0.290	0.008	0.049	0.001
quru-GA-0M05	201	174	21	18	6	3	0.766	0.754	0.798	0.808	0.008	0.026	0.013
quru-GA-0M07	201	174	9	12	0	3	0.732	0.766	0.770	0.829	0.043	0.040	0.018
quru-GA-1C08	200	173	16	15	1	0	0.723	0.795	0.800	0.819	0.019	0.110	0.017
MSQ4	201	174	18	27	1	10	0.807	0.780	0.899	0.919	0.008	0.017	0.031
MSQ13	200	172	12	12	2	2	0.721	0.703	0.808	0.798	0.018	0.055	0.044
MSQ16	199	171	15	19	0	4	0.855	0.799	0.894	0.809	0.051	0.025	0.015
ssrQrZAG7	201	174	20	22	0	2	0.915	0.954	0.911	0.933	0.019	0.023	0.013
ssrQrZAG87	200	173	23	36	0	13	0.770	0.919	0.818	0.951	0.061	0.044	0.007
ssrQrZAG96	201	174	24	22	5	3	0.852	0.874	0.928	0.884	0.025	0.018	0.020
ssrQrZAG101	201	172	21	24	2	5	0.794	0.930	0.842	0.918	0.046	0.110	0.025
ssrQrZAG102	201	173	38	42	2	6	0.937	0.954	0.967	0.963	0.004	0.021	0.011
ssrQrZAG112	201	174	16	17	2	3	0.825	0.711	0.841	0.759	0.097	0.039	0.025
bcqm42F	201	172	11	9	2	0	0.758	0.596	0.745	0.623	0.031	0.043	−0.001
All	201	174	323	357	32	66	0.761	0.729	0.801	0.764	0.049**	0.046**	0.019**

QL: seven populations of *Q. liaotungensis*; QM: six populations of *Q. mongolica*; N: number of individual analyzed; *A_n_:* number of alleles over all populations for each species; *A_P_*: number of private alleles; *H_O_*: observed heterozygosity; *H_E_*: expected heterozygosity;

**: p<0.01.

The application of the four AFLP primer combinations to 419 oak individuals resulted in 194 non-monomorphic markers (1%<frequency<99%), of which 133 (69%) were polymorphic at the 5% level in *Q. liaotungensis* and 124 (64%) in *Q. mongolica*. The unbiased estimate of genetic diversity (*H_j_*) for each population varied from 0.204 to 0.234 (Table 1). The vast majority of the diversity is partitioned within-population in both species. The levels of diversity within each species, either at the population (*H_w_*) or the whole-sample level (*H_t_*), were strikingly similar (Table 4).

**Table 4:** Comparison of genetic diversity and differentiation between *Quercus liaotungensis* and *Q. mongolica* based on 194 AFLP markers.

Populations	N	*Ht*	*Hw*	SE(*Hw*)	*Hb*	SE(*Hb*)	*F*_ST_	lower 99% *F*_ST_	upper 99% *F*_ST_
Between species	2	0.234	0.212	0.005	0.022	<0.001	0.093	−0.001	0.003
*Q. liaotungensis*	7	0.227	0.217	0.004	0.010	0.001	0.044	−0.005	0.003
*Q. mongolica*	6	0.216	0.213	0.002	0.003	<0.001	0.014	−0.006	0.002

N: number of populations; *Ht*: total diversity; *Hw*: average diversity within population; *Hb*: average diversity between populations; *F*_ST_: differentiation between populations.

The Weir and Cockerham's (1984) estimator of genetic differentiation between *Q. liaotungensis* and *Q. mongolica* was low but significant across all SSR loci (*θ__inter_* = 0.049, *p*<0.01). At most SSR loci, the interspecific *θ* was low, with 8 of the 19 loci displaying values <0.02 and only 3 having values >0.10 (Table 3). The differentiation among populations within species was lower though also significant for both species [*θ*__*intra*_ = 0.046 (*p*<0.01) for *Q. liaotungensis*; *θ*__*intra*_ = 0.019 (*p*<0.01) for *Q. mongolica*]. When the analysis was restricted to ‘pure’ individuals (i.e. individuals assigned at >0.8 to one of the two species), the interspecific differentiation across all SSR loci was larger (*θ__pure inter_* = 0.067, *p*<0.01). The differentiation among populations within species was also higher [*θ*__*pure intra*_ = 0.049 (*p*<0.01) for *Q. liaotungensis*; *θ*__*pure intra*_ = 0.020 (*p*<0.01) for *Q. mongolica*]. The genetic differentiation between *Q. liaotungensis* and *Q. mongolica* across the 194 AFLP markers was low but significant (*F*_ST_*inter*_ = 0.093, *p*<0.01; Table 4). The differentiation among populations within species was lower but significant for both species [*F*_ST_*intra*_ = 0.044 (*p*<0.01) for *Q. liaotungensis*; *F*_ST_*intra*_ = 0.014 (*p*<0.01) for *Q. mongolica*; Table 4]. When the analysis was restricted to ‘pure’ individuals, the interspecific differentiation across the 194 AFLP markers became more pronounced (*F*_ST_*pure inter*_ = 0.131, *p*<0.01) and was much higher than the differentiation among populations within species [*F*_ST_*pure intra*_ = 0.042 (*p*<0.01) for *Q. liaotungensis*; *F*_ST_*pure intra*_ = 0.010 (*p*<0.01) for *Q. mongolica*]. The higher differentiation within *Q. liaotungensis* than within *Q. mongolica* was mainly caused by the divergence of THl and of ZW and WA, the two allopatric *Q. liaotungensis* populations (see Table 5).

**Table 5:** Pairwise *θ* (Weir and Cockerham, 1984) based on 19 SSRs (above the diagonal) and pairwise *Φ*_PT_ (a statistic analogous to *F*_ST_, Peakall *et al*., 1995) based on 194 AFLPs (below the diagonal).

	ZW	WA	CF2	FS	SPl	THl	NAl	HH	MR	Dan	SPm	THm	NAm	Dmy	Dtt
ZW		**0.036** **	**0.050** **	**0.052** **	**0.036** **	**0.078** **	**0.055** **	0.112 **	0.120 **	0.096 **	0.074 **	0.097 **	0.095 **	0.029 **	0.066 **
WA	**0.022** **		**0.061** **	**0.041** **	**0.032** **	**0.086** **	**0.059** **	0.124 **	0.120 **	0.109 **	0.077 **	0.103 **	0.100 **	0.047 **	0.074 **
CF2	**0.111** **	**0.097** **		**0.039** **	**0.025** **	**0.070** **	**0.029** **	0.067 **	0.077 **	0.059 **	0.046 **	0.065 **	0.056 **	0.045 **	0.034 **
FS	**0.089** **	**0.070** **	**0.050** **		**0.007** *	**0.052** **	**0.023** **	0.087 **	0.100 **	0.076 **	0.043 **	0.071 **	0.069 **	0.055 **	0.052 **
SPl	**0.094** **	**0.070** **	**0.049** **	**0.016** **		**0.047** **	**0.011 NS**	0.063 **	0.074 **	0.052 **	0.027 **	0.055 **	0.044 **	0.034 *	0.032 **
THl	**0.142** **	**0.118** **	**0.112** **	**0.091** **	**0.086** **		**0.044** **	0.105 **	0.110 **	0.087 **	0.061 **	0.073 **	0.086 **	0.076 **	0.067 **
NAl	**0.108** **	**0.097** **	**0.038** **	**0.038** **	**0.026** **	**0.092** **		**0.043** **	**0.051** **	**0.037** **	**0.017** **	**0.032** **	**0.027** **	0.044 **	0.026 **
HH	0.233 **	0.243 **	0.151 **	0.165 **	0.137 **	0.188 **	0.106 **		**0.035** **	**0.021** **	**0.017** **	**0.021** **	**0.014** **	0.079 **	0.031 **
MR	0.260 **	0.270 **	0.166 **	0.192 **	0.165 **	0.218 **	0.119 **	**0.043** **		**0.032** **	**0.036** **	**0.021** **	**0.022** **	0.091 **	0.037 **
Dan	0.238 **	0.238 **	0.144 **	0.166 **	0.135 **	0.182 **	0.091 **	**0.035** **	**0.046** **		**0.010** **	**0.013** **	**0.013** **	0.065 **	0.019 **
SPm	0.168 **	0.169 **	0.081 **	0.092 **	0.075 **	0.133 **	0.042 **	**0.036** **	**0.043** **	**0.017** **		**0.010** **	**0.009** **	0.044 **	0.013 **
THm	0.234 **	0.236 **	0.147 **	0.161 **	0.132 **	0.170 **	0.094 **	**0.029** **	**0.044** **	**0.018** **	**0.012** **		**0.009 NS**	0.065 **	0.017 **
NAm	0.217 **	0.225 **	0.121 **	0.148 **	0.113 **	0.170 **	0.072 **	**0.022** **	**0.041** **	**0.025** **	**0.012** *	**0.012** *		0.066 **	0.022 **
Dmy	0.084 **	0.081 **	0.044 **	0.051 **	0.037 **	0.114 **	0.026 **	0.137 **	0.152 **	0.110 **	0.053 **	0.106 **	0.096 **		0.035 **
Dtt	0.129 **	0.128 **	0.070 **	0.072 **	0.043 **	0.102 **	0.036 **	0.062 **	0.080 **	0.053 **	0.017 **	0.045 **	0.044 **	0.036 **	

*: 0.01<*p*<0.05,

**: *p*<0.01,

NS: Non-Significant. Pairwise genetic differentiation within species is indicated in bold.

Pairwise population *θ* and *Φ*_PT_ values with corresponding significant levels are presented in Table 5. Significant genetic differentiation was found among all population pairs in the AFLP data set and all but two (SPl and NAl, THm and NAm) in the SSR data set. For both AFLP and SSR data sets, genetic differentiation was low between all *Q. mongolica* population pairs; whereas among *Q. liaotungensis* populations, the THl, ZW and WA population were significantly differentiated from other *Q. liaotungensis* populations.

### Genetic distance-based analysis

**Figure 2:**
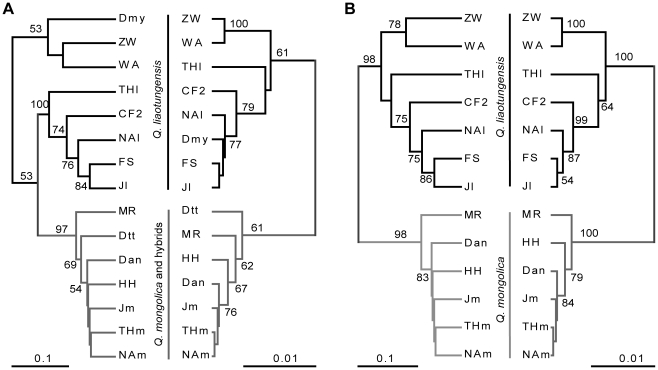
Dendrograms of oak populations based on genetic distance. (A) With all populations. (B) After excluding the two populations from the Dongling Mountain region. In both panels, left dendrograms are based on genetic distance of 19 SSR loci and right dendrograms are based on genetic distance at 194 AFLP bands. The dendrograms were computed using a UPGMA approach implemented in PHYLIP. Numbers are bootstrap support values.

Overall, the two UPGMA trees based on SSR and AFLP data sets were highly congruent (Fig. 2A). All typical *Q. mongolica* populations and the Dtt population shared a common node with a bootstrap value over 60% in both trees; all *Q. liaotungensis* populations shared the other common node in the AFLP tree. In the SSR tree, *Q. liaotungensis* populations were separated in two groups: the sympatric group vs the allopatric group; the sympatric *Q. liaotungensis* group, which is comprised of populations from northeast China and Chifeng, was more closely related to *Q. mongolica* populations than to the allopatric *Q. liaotungensis* group comprised of ZW, WA and Dmy populations. When the Dtt and Dmy populations were excluded from the analysis, the SSR and AFLP trees had exactly the same topology, with the two species separated at very strong bootstrap support (98% for SSR and 100% for AFLP; Fig. 2B).

**Figure 3:**
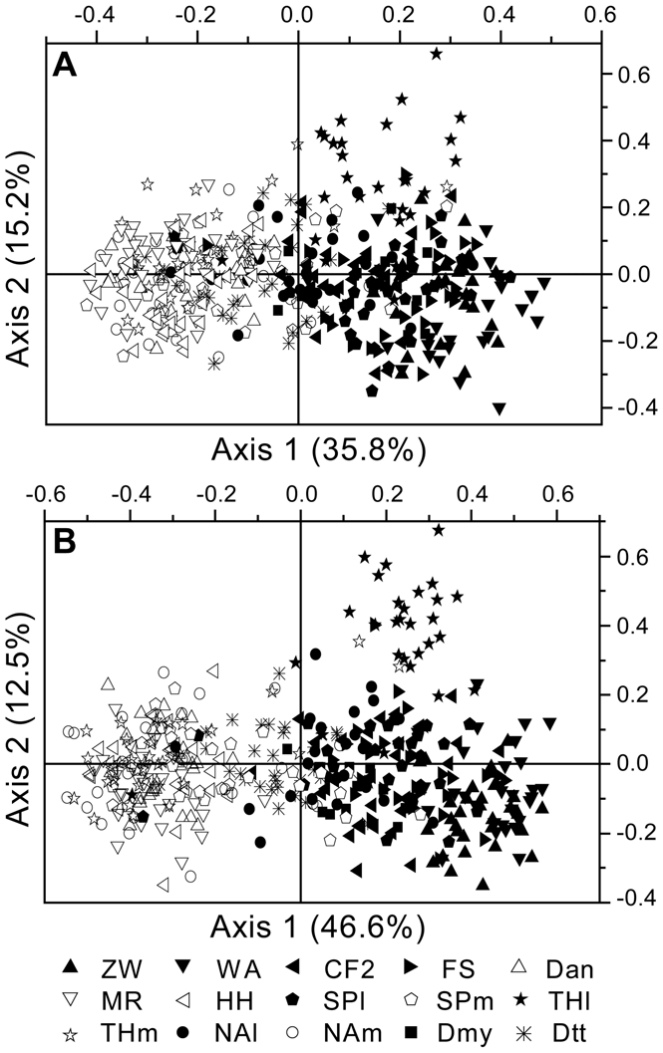
PCo plots of pairwise individual genetic distances Φ at SSR (A) and AFLP (B) markers. Filled symbols: *Quercus liaotungensis*; open symbols: *Q. mongolica*; radial symbols: intermediate individuals from population Dtt.

The results of the PCo for the pairwise individual genetic distances are presented in Fig. 3. The two first PCo-axes of the SSR (Fig. 3A) and the AFLP (Fig. 3B) plot accounted for about 51% and 59% of the variation, respectively. Both SSR and AFLP data grouped individuals from *Q. liaotungensis* and *Q. mongolica* populations separately, but AFLP distinguished the individuals of the two groups slightly better. Most individuals from the Dmy population had a close relationship with *Q. liaotungensis*, and most individuals from Dtt had intermediate positions. The AFLP plot generally showed a higher resolution, and species groups appeared more distinct than in the SSR plot.

### Bayesian cluster results

**Figure 4:**
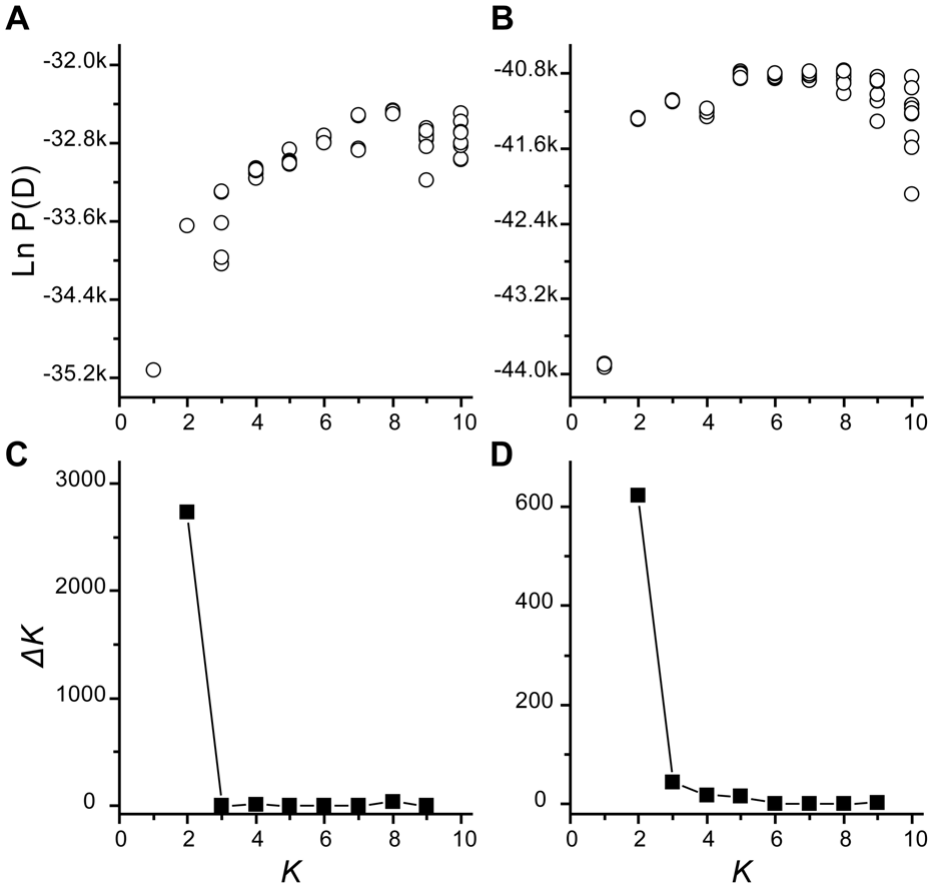
Indication of the most likely number of clusters in the STRUCTURE analysis. Both the estimated logarithmic probability (panels A and B) and magnitude of *ΔK* (panels C and D) as a function of *K* suggested the existence of two clusters. Results are from 10 replicates for each of 1≤*K*≤10 with both SSR (panels A and C) and AFLP markers (panels B and D).

**Figure 5:**
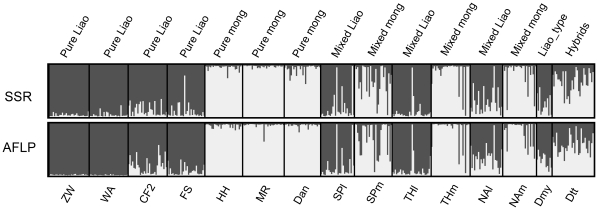
STRUCTURE results for two clusters with no prior population knowledge. Results are based on 19 SSR loci (above) or 194 AFLP markers (below). Each individual is represented by a thin vertical line. Black lines separate individuals of different populations. Populations are labelled below the figure with their regional affiliations. Previous population morphological information is provided above the figure. (Pure Liao: pure *Quercus liaotungensis*; Pure Mong: pure *Q. mongolica*; Mixed Liao and Mixed Mong: *Q*. *liaotungensis* and *Q*. *mongolica* from mixed forests, respectively.

**Figure 6:**
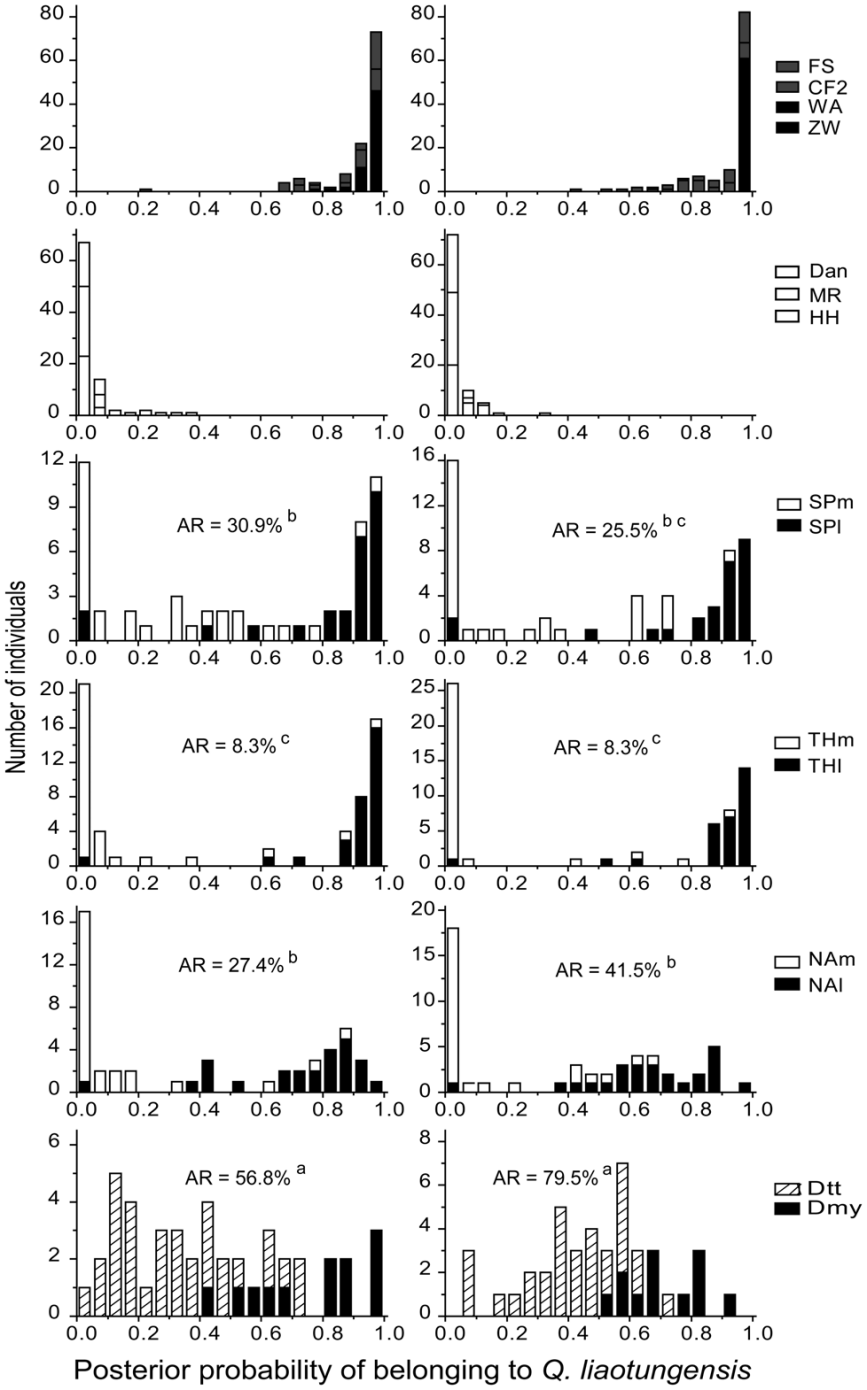
Distribution of ancestry estimates in each study site. The ancestry is based on posterior probability of belonging to *Quercus liaotungensis* at *K* = 2 in the STRUCTURE analysis using 19 SSR loci (left) or 194 AFLP markers (right). AR =  admixture rate according to the STRUCTURE assignment analysis, with the superscript a, b and c indicating statistically significant differences.

The STRUCTURE output for both SSR and AFLP data sets suggested the existence of two clusters. The AFLP and SSR data sets gave similar results, with *K* = 2 being considerably more likely than *K* = 1, while *K*≥3 being only slightly more likely (Fig. 4A and B). *ΔK* distribution further supported the choice of *K* = 2, showing distinct modal distribution at *K* = 2 (Fig. 4C and D). The cluster patterns for the two data sets were also very similar; moreover, the two genetically distinct clusters corresponded well to our morphological assignment of populations to *Q. liaotungensis* and *Q. mongolica* (Fig. 5 and Fig. 6).

**Figure 7:**
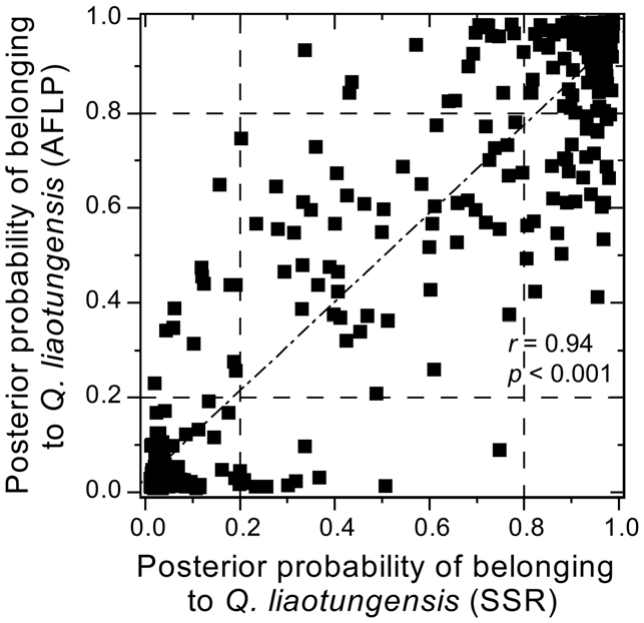
Correspondence between the SSRs and AFLPs assignment results in STRUCTURE analysis. Each black square represents a single individual. Dashed lines denote the threshold of the successful assignment to either cluster (posterior probability <0.2: *Quercus mongolica*; posterior probability >0.8: *Q. liaotungensis*; 0.2< posterior probability <0.8: hybrids).

More specifically, SSRs results indicated that 42% of all individuals had a posterior probability >0.8 of belonging to *Q. liaotungensis*, and 38% to *Q. mongolica*; the corresponding values based on AFLPs are 40% and 38% (Table 6). The assignment results based on the two types of markers were highly congruent. The mean posterior probability of belonging to *Q. liaotungensis* is 0.52 for SSRs and 0.51 for AFLPs. The Pearson correlation coefficient between posterior probabilities for SSRs and for AFLPs was high and significant (*r* = 0.94, *p*<0.001, Fig. 7). The correlation remained significant but dropped sharply after excluding genotypes assigned to pure species with both approaches (*r* = 0.52, *p*<0.001). With both SSR and AFLP datasets, the majority of individuals from pure sites were assigned to their respective clusters, except in Chifeng, where the percentage of hybrids reached 30% (according to SSR) and 37% (according to AFLP). Most individuals from the three mixed sites where both species are present were also successfully assigned to their respective clusters, with only a few individuals being assigned to the alternative cluster (Siping: 7.3% and 5.5%; Tonghua: 5.0% and 3.3%; Ning'an: 3.9% and 2.0%, for SSR and AFLP respectively). The percentage of hybrids in the three mixed sites varied among different sites (Table 6). Tonghua had the fewest hybrids (8.3% and 8.3% for SSR and AFLP, respectively) as compared to Siping (SSR: 30.9%, *χ* ^2^ = 9.5, *p* = 0.002; AFLP: 25.5%, *χ* ^2^ = 6.1, *p* = 0.014) and Ning'an (SSR: 27.4%, *χ* ^2^ = 7.1, *p* = 0.008; AFLP: 45.1%, *χ* ^2^ = 17.8, *p*<0.001), whereas the percentage of hybrids in Dongling Mountain (56.8% for SSR and 79.5% for AFLP) was significantly higher than at any of the three mixed sites (SSR: *χ* ^2^>6.7, *p*<0.01 for all comparisons; AFLP: *χ* ^2^>11.8, *p*≤0.001 for all comparisons). Most hybrids there were from Dtt population. In contrast, a total of 7 (according to SSR) and 4 (according to AFLP) of the 12 Dmy individuals had a posterior probability >0.8 of belonging to the *Q. liaotungensis* cluster.

**Table 6:** Number (and percentage) of pure species and hybrid oaks as assigned by the STRUCTURE software in the different studied sites.

			SSR			AFLP		
Study site	Population ID	N	QL	QM	Hybrids	QL	QM	Hybrids
Ziwu	ZW	31	31 (100.0%)	-	-	31 (100.0%)	-	-
Wuan	WA	30	29 (96.7%)	-	1(3.3%)	30 (100.0%)	-	-
Chifeng	CF2	30	21 (70.0%)	-	9 (30.0%)	19 (63.3%)	-	11 (36.7%)
Fushun	FS	29	24 (82.8%)	-	5(17.2%)	25 (86.2%)	-	4 (13.8%)
Heihe	HH	29	-	28 (96.6%)	1 (3.4%)	-	29 (100.0%)	-
Mao'ershan	MR	32	-	32 (100.0%)	-	-	31 (96.8%)	1 (3.2%)
Dandong	Dan	28	-	24 (85.7%)	4 (14.3%)	-	28 (100.0%)	-
Siping	SPl+SPm	55	22 (40.0%)	16 (29.1%)	17 (30.9%) b	22 (4.0%)	19 (34.5%)	14 (25.5%) bc
Tonghua	THl+THm	60	29 (48.3%)	26 (43.3%)	5 (8.3%) c	28 (46.7%)	27 (45.0%)	5 (8.3%) c
Ning'an	NAl+NAm	51	14 (27.4%)	23 (45.1%)	14 (27.4%) b	8 (15.7%)	20 (39.2%)	23 (45.1%) b
Dongling Mountain	Dtt+Dmy	44	7 (13.7%)	12 (25.0%)	25 (56.8%) a	4 (9.1%)	5 (11.4%)	35 (79.5%) a
Total		419	177 (42.2%)	161 (38.4%)	81(19.3%)	167 (39.9%)	159 (37.9%)	93 (22.2%)

N: number of sampled oaks; QL: *Quercus liaotungensis*; QM: *Q. mongolica*;

a b c indicated statistic significant (*p*<0.01) differences for the hybridization rate comparison between different sites.

### Loci under disruptive selection

**Figure 8:**
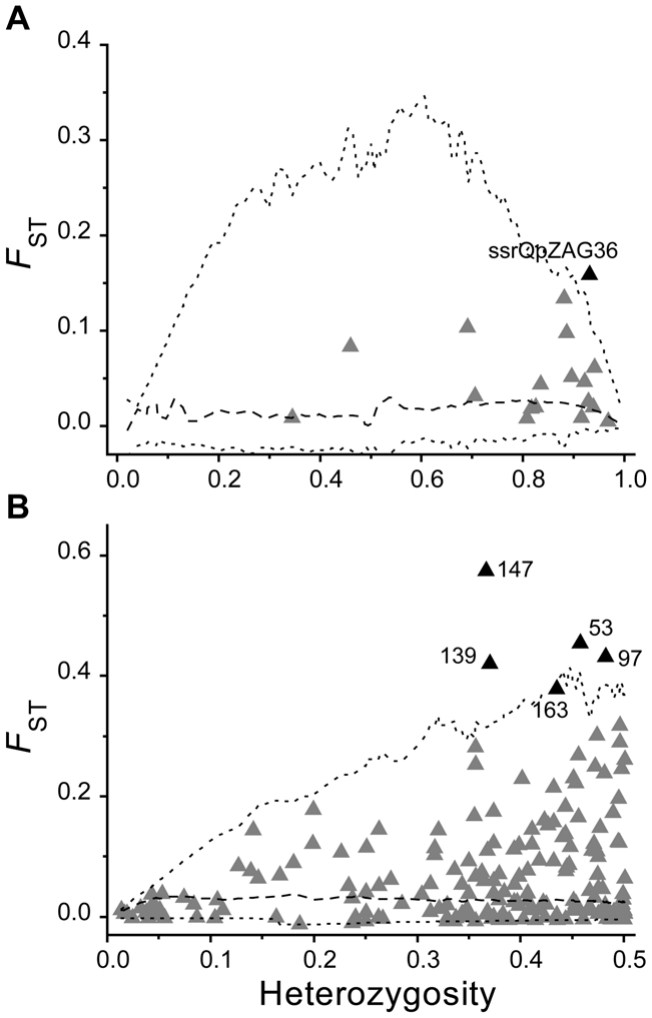
Distribution of per-locus *F*_ST_ values (differentiation between *Quercus liaotungensis* and *Q*. *mongolica*) against heterozygosity. Each triangle represents a SSR (panel A) or AFLP (panel B) marker. The black triangles above the upper line are classified as outliers potentially under divergent selection. Dotted lines denote 99th and 1th quantiles estimated from simulation, and dashed lines denote the medians.

Simulation analysis revealed that one SSR locus (ssrQpZAG36) and five AFLPs are significant outliers on a plot of interspecific *F*_ST_ against heterozygosity (Fig. 8). Therefore they are likely to be either under disruptive selection or linked to a locus under selection [68], [69]. Most of these outlier loci were also identified as outliers in the separate pairwise interspecific comparisons within the three mixed populations (Fig. S1). The AFLP outlier locus with the highest *F*_ST_ value (0.57) had a large difference in allelic frequency between *Q. liaotungensis* (95%) and *Q. mongolica* populations (14%). However, the interspecific *F*_ST_ of most SSR and AFLP loci exhibited very low values, suggesting substantial genetic exchanges at loci unlikely to be under disruptive selection (Fig. 8).

## Discussion

### Species status

The taxonomic status of closely related oak species has long been an issue of controversy because of continuous variation in morphological, ecological, and genetic traits due to interspecific hybridization and/or shared ancient polymorphisms [11], [12], [18], [31], [70]. This is the case for *Q. liaotungensi*s and *Q. mongolica*, dominant members of warm temperate forests in northern China and the surrounding regions. A previous molecular study of the two oaks inferred strong gene flow between species and did not resolve the species status issue [43]. The present study revealed a clear differentiation of the Chinese oak gene pool into two entities corresponding to *Q. liaotungensis* and *Q. mongolica*. Acorn cupule and trunk bark characteristics were helpful to discriminate the species; in contrast, the number of lateral veins did not appear helpful for taxonomic purposes.

Although clustering based on population genetic distance relies on a priori classification of individuals or populations, it can provide useful insights. In our study, high bootstrap values and consistent topology between SSR and AFLP trees (Fig. 2) indicated that the differentiation based on the acorn cupule and bark morphology was associated with a clear and stable genetic differentiation at molecular markers. In particular, at the three mixed sites, populations were clustered into two groups according to species rather than to geographic origin. Two analyses that do not rely on a priori classifications (Bayesian clustering analysis using the software STRUCTURE and principal coordinate analysis based on individual pairwise distances) were then carried out. Both revealed a separation of the individuals into two groups that correspond well with the taxonomic species. Comparable results in the European white oak complexes have been obtained in both SSR [24] and AFLP surveys [71]. These analyses established the species status of sessile (*Q. petraea*) and pedunculate oaks (*Q. robur*). However, the bootstrap support for each group in our SSR study was not 100%, as in Muir *et al.* [24], for the following reasons. First, we did not get detailed morphological data for each individual. The identification based on one or two morphological characteristics might have led to false classification, thus ultimately reducing the support values. Second, genotypic and phenotypic mismatch might exist for a few individual trees, especially for hybrids [25], which could reduce the genetic distance between species.

The most likely clustering in STRUCTURE (with *K* = 2) also revealed that the taxonomical signal was much stronger than the geographic signal. The Bayesian clustering approach that was developed to identify genetic structure in the mixed populations has often been applied to test for the presence of a taxon without assigning individuals to a predefined group [25], [27], [29], [72]. This approach allows evaluation of the statistical significance of clusters. Moreover, the origin of individuals can be inferred by calculating the probability that individual multilocus genotypes belong to different genetic clusters, or alternatively, are hybrid in origin [62], [63]. Without detailed individual morphological data, our multilocus analysis assigned most individuals from both pure and mixed sites into groups recognizable as the two species, *Q. liaotungensi*s and *Q. mongolica*, with only a few mismatched individuals and hybrids. When each of three pairs of mixed populations was analyzed separately, AFLP could also categorize individuals into the respective species (Fig. 10 in [73]). These results suggest that *Q. liaotungensi*s and *Q. mongolica* occur as distinct clusters of genotypes even where they co-occur locally. One population that we have to mention is Dmy. Its nearest population, Dtt, consisted mostly of hybrid individuals; however, STRUCTURE analysis suggested that most individuals in the Dmy population were *Q. liaotungensi*s, consistent with their morphology (smooth-cupules acorns).

The existence of outlier loci indicated that, although *Q. liaotungensis* and *Q. mongolica* form hybrids, they remain generally distinct at some genomic regions when in sympatry. *Q. robur* and *Q. petraea* have also been shown to represent separate species using both morphological data [74] and molecular markers [24], [71]; however, until now, no single marker has been identified that can differentiate between them at the individual tree level. With 176 polymorphic AFLP markers, Coart *et al.* [71] classify *Q. petraea* and *Q. robur* populations into two main groups with very high bootstrap support (100%), in agreement with their taxonomic status. However, in their study, only one marker displays a difference in frequency up to 71%. With comparable polymorphic markers, we identified one fragment with a difference in frequency between *Q. liaotungensis* and *Q. mongolica* of 81% (95% vs 14%); this difference would be even higher if the individuals suggested by STRUCTURE as having been misidentified had been corrected, suggesting that the two species studied are at least as valid as the well-investigated *Q. robur* and *Q. petraea*.

### Comparison between AFLP markers and SSR loci

Although AFLP and SSR markers have been widely used in describing interspecific differentiation in oaks, direct comparisons of genotype assignment using AFLP and SSR are still lacking. In our study, the interspecific differentiation estimated by *F*_ST_ between *Q. liaotungensi*s and *Q. mongolica* was slightly higher for AFLP (*F*_ST_inter_ = 0.093) than for SSR (*θ__inter_* = 0.049), in line with findings for *Q. robur* and *Q. petraea* by Mariette *et al*. [75] (but see [76]). Our assignment of all individuals into groups with the STRUCTURE program gave very similar results with the AFLP and the SSR data sets, as did the PCo plot based on individual distances and the topology identified in the UPGMA trees. However, the AFLP markers were more powerful than the SSR loci in discriminating the origin of individuals from different species. Indeed, the AFLP multilocus Bayesian cluster analysis showed higher assignment success than SSR loci: (1) For individuals from allopatric pure sites, the posterior probability of belonging to their respective species measured with AFLP markers was generally higher than the probability measured with SSR loci; (2) when we did Bayesian cluster analysis separately for each of the three pairs of mixed populations (THl vs THm, SPl vs SPm, and NAl vs NAm), AFLP markers could successfully assign individuals into distinct species for all three pairs of mixed populations, whereas SSR loci succeeded only in Tonghua location (THl vs THm). Furthermore, a larger proportion of variation was explained by the two first axes of the PCo with the AFLPs than with the SSRs, and the AFLP plots gave higher resolution and distinguished the individuals of different species slightly better (Fig. 3). Better genome coverage due to the larger number of loci is likely responsible for the higher resolution with the AFLP dataset compared to the SSR dataset.

Although the AFLP dataset provided higher resolution than the SSR dataset, both types of markers provided comparable and unbiased results. The overall mean species assignments were very close with each type of markers and the correlation between individual assignment values for the two types of data was high, although it was much lower for admixed individuals. These results indicate that similar conclusions regarding species delimitation can be arrived at using independent marker sets but also show that precise estimates of individual introgression rates require sizeable genome-wide datasets.

### Hybridization and hybrid zones

Our investigation of *Q. liaotungensis* and *Q. mongolica* suggested that hybridization occurs between the two species in their sympatric range. First, the approximate Bayes factors (ratio of the estimated marginal likelihood of the admixture model to that of the non-admixture model) was greater than 100:1 for both the SSR and AFLP data sets, which could be considered as ‘decisive’ [77]. Second, the analyses of the AFLP and SSR datasets independently pointed to the existence of a subset of admixed individuals (with a posterior probability <0.8 of belonging to either cluster). Third, the interspecific *F*_ST_ values were remarkably variable across markers, with many loci displaying low *F*_ST_ values (<0.02). Fourth, past hybridization between the two species is suggested by the extensive sharing of cpDNA in North China and Northeast China [73].

Bayesian assignment using multilocus genotype suggests that the proportion of hybrids varies in different geographic contact sites of *Q. liaotungensis* and *Q. mongolica* (Table 6 and Fig. 6). The hybrid zones from Northeast China consisted largely of genotypes resembling the parental forms, and thus constitute bimodal hybrid zones, suggestive of well-developed (although incomplete) reproductive isolation [3], [78]. In contrast, the Dtt hybrid zone in North China was composed largely of recombinant individuals, corresponding to a unimodal hybrid zone or hybrid swarm, suggestive of incomplete reproductive isolation.

The variation in hybridization patterns between these two species in different parts of the range is of great interest. Such patterns have been reported in several plant species and many possible causes for this variation have been discussed. First, the degree of actual intermixing might not be uniform in different contact zones, resulting in differential hybridization and introgression [e.g. 27]. Second, differences in relative abundance of each species locally could affect rates and direction of introgression [34]. Third, the presence of open, intermediate, marginal or disturbed habitats could promote hybridization, as reported previously in oaks [12], [79]–[84]. In fact, during range expansions, both asymmetric population size and the presence of open habitats could result in a short-term increase of the proportion of hybrids [85], [86]. Another possibility that has been much less explored is *reproductive character displacement*, also called reinforcement of reproductive barriers, a pattern of greater divergence of a trait between closely related taxa in areas of sympatry than in areas of recent contact following allopatric divergence [87].

STRUCTURE assignment results suggest that hybrid frequency in Northeast China varies among the three mixed sites, ranging from 8% to 31–45%, depending on marker type. This proportion of hybrids is comparable to that of the European white oak complex (11–30%, see [34]). *Q. liaotungensis* and *Q. mongolica* shared most nuclear alleles, with only a few low-frequency private alleles being identified in our studied populations. The introgression direction differed depending on the location of hybrid zones. The hybrid zone in Siping suggests bidirectional introgression, whereas in Ning'an hybrids had a genetic composition closer to *Q. liaotungensis* (Fig. 6), indicating directional introgression. The directional introgression in Ning'an mixed forest might be due to the relatively low abundance of *Q. liaotungensis* in this region (see [34]). According to the *Higher Plants of China* ([38], see Fig. 1), and as confirmed by our own field observation, Ning'an represents the northeastern edge of the present distribution of *Q. liaotungensis.* The heterogeneous level of admixture and introgression direction in Northeast China might also relate to the precise location of oaks' glacial refugium and subsequent recolonization processes. Further studies are needed to investigate the role of hybridization and introgression during recolonization processes of Chinese oaks since the last glacial maximum [88].

Alternatively, the differences between contact zones might be explained by geographically variable natural selection against hybrids. The infrequency of hybrids in Northeast China might result from their having lower fitness than the parental species. In fact, in Tonghua, where rates of introgression were very low, more loci under disruptive selection were identified in comparison with the two other mixed sites (Fig. S1), even after accounting for misclassified individuals (analysis not shown). The existence of loci potentially under disruptive selection suggests directional selection on a subset of loci between *Q. liaotungensis* and *Q. mongolica* genomes in mixed sites of Northeast China. However, hybrids might exhibit an increased fitness in an intermediate or altered (mostly anthropogenic) environment uncharacteristic of either parental species, such as in Dtt, a touristic place located in the suburb of Beijing. Further artificial pollination and transplant experiments are needed to test the mechanism by which reproductive isolation and habitat selection affects species delimitation of *Q. liaotungensis* and *Q. mongolica*.

Finally, to evaluate the hypothesis of reproductive character displacement, it will be necessary to reconstruct the history of the two species in their different contact zones. For instance, long time co-occurrence of *Q. liaotungensis* and *Q. mongolica* in Northeast China could have reinforced their reproductive isolation barrier, resulting in stronger barriers than in the new contact zone between *Q. liaotungensis* and *Q. mongolica* in northern China.

### Conclusions

Our molecular analysis led to the conclusion that *Q. liaotungensi*s and *Q. mongolica* have maintained distinct gene pools, even in mixed stands, and should be considered as discrete taxonomic units, despite the existence of interspecific hybridization. Results based on SSRs and AFLPs were highly congruent, indicating that the conclusions reached regarding species delimitation and hybridization are of general value. Interestingly, hybridization rates were not uniform in different contact zones. More work is needed to tease apart the mechanisms underlying this heterogeneity of interspecific genetic differentiation across the species' ranges.

## Supporting Information

Figure S1 Distribution of per-locus interspecific FST values against heterozygosity in each study site.
(TIF)
