# Supplementary figures and images for: Exploring Species Limits in Two Closely Related Chinese Oaks
Source: PLoS One. 2010 Nov 30;5(11):e15529. doi: 10.1371/journal.pone.0015529 (PMC2994836; doi:10.1371/journal.pone.0015529)

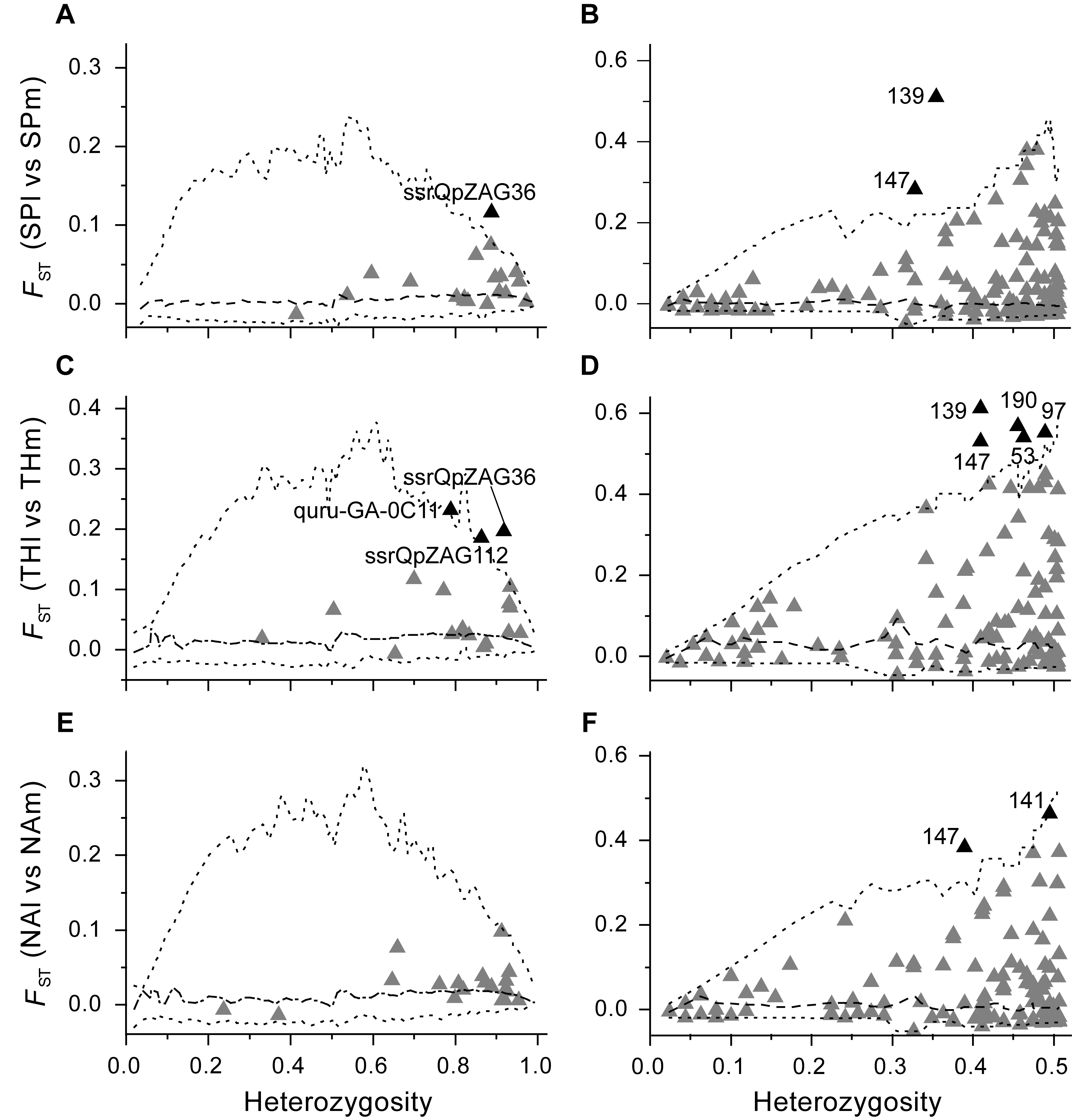

Supplement: Figure S1 — Distribution of per-locus interspecific FST values against heterozygosity in each study site. (TIF) [file pone.0015529.s001.tif]
